# Pectoralis major tendon transfer for management of subscapularis failure after anatomic total shoulder arthroplasty: technique and results

**DOI:** 10.1016/j.xrrt.2022.05.001

**Published:** 2022-06-09

**Authors:** Gary Updegrove, Jacquelyn Kohler, Padmavathi Ponnuru, April D. Armstrong

**Affiliations:** a Department of Orthopaedics and Rehabilitation, Penn State Hershey Bone and Joint Institute, Penn State Milton S. Hershey Medical Center, Hershey, PA, USA; b Penn State Milton S. Hershey Medical Center, Penn State College of Medicine, Hershey, PA, USA

**Keywords:** Shoulder, Arthroplasty, Revision, Subscapularis, Pectoralis, Transfer

## Abstract

**Background:**

Subscapularis tendon failure after anatomic total shoulder arthroplasty can lead to pain, dysfunction, and early component failure. The purpose of this study was to report on the results of pectoralis major tendon transfer for treatment of irreparable subscapularis tendon failure in the setting of prior shoulder arthroplasty.

**Methods:**

Patients who underwent pectoralis major muscle transfer for treatment of subscapularis failure in the setting of prior total shoulder arthroplasty or hemiarthroplasty were included in the study. The entirety of the pectoralis major tendon was transferred superficial to the conjoined tendon and placed lateral to the bicipital groove.

**Results:**

Eight patients were included in the study. All 7 patient who experienced pain in their shoulder had improvement in their pain postoperatively. Those patients with preserved active motion were able to regain that motion postoperatively. Radiographically, anterior translation was found to be temporarily improved; however, anterior instability would later recur in most cases, though this did not correlate with increased pain or decreased function.

**Discussion:**

Management options ranging from continued observation, revision repair, pectoralis muscle transfer, or revision to reverse total shoulder arthroplasty should be considered in a setting of subscapularis failure after shoulder arthroplasty. Decision-making should be based on physiological age of the patient as well as symptoms present as well as the position and stability of the arthroplasty components.

**Conclusion:**

Pectoralis muscle transfer can provide pain relief, improve subjective instability, and preserve function in physiological young patients with an irreparable subscapularis who have well-positioned and well-fixed anatomic shoulder arthroplasty components.

Failure of healing of the subscapularis tendon repair after anatomic total shoulder arthroplasty is a known complication that can lead to pain, early component failure, and ultimately revision surgery.^15^^,^^16^^,^^23^^,^^26^ Though this problem has led to some authors to trial exposure for total shoulder arthroplasty utilizing a subscapularis sparing approach; most anatomic total shoulder arthroplasty procedures are performed utilizing a technique to reflect the subscapularis either by tenotomizing the tendon, peeling the tendon from the lesser tuberosity, or performing a lesser tuberosity osteotomy.^1^^,^^16^^,^^17^^,^^20^^,^^21^^,^^23^ Subscapularis insufficiency following shoulder arthroplasty may be asymptomatic, noted incidentally on physical examination or postoperative radiographs, or could lead to pain, shoulder dysfunction, instability, or early component failure.^16^^,^^17^

When identified acutely postoperatively, primary repair of the tendon is indicated and often demonstrates favorable results.^9^^,^^17^ In patients where the tear or insufficiency discovery is delayed, direct repair of the tendon may not be possible. This could be due to significant retraction of the tendon, poor tendon quality, or fatty muscle atrophy and degeneration.

Reverse shoulder arthroplasty is a dependable option for management of patients with subscapularis deficiency after shoulder arthroplasty; however, this involves significant exposure and the need for component exchange.^19^ In many cases, this will involve removal of a well-fixed glenoid component to convert to baseplate fixation, as well as possible revision of the humeral stem. Though revision to reverse shoulder arthroplasty can improve pain, function, and quality-of-life measures, postoperative range of motion and patient-reported outcomes are lower than those seen after primary shoulder arthroplasty.^10^^,^^24^ Convertible shoulder platform systems may have the potential to make these revisions easier with the ability to convert from anatomic to reverse components while retaining the stem and, in some cases, the glenoid baseplate fixation; however, in some cases, the stem, though convertible, may need revision due to positioning.^2^^,^^12^^,^^13^

A less-invasive option than revision to reverse total shoulder arthroplasty for management of subscapularis insufficiency or failure is transfer of the pectoralis major tendon.^11^^,^^18^ Most commonly performed for irreparable subscapularis tendon tears in native joint anatomy, this procedure has also been utilized in the setting of anatomic total shoulder arthroplasty and hemiarthroplasty, though with less predictable results.^18^^,^^23^

We present a surgical technique for pectoralis major tendon transfer in the setting of anatomic total shoulder arthroplasty and hemiarthroplasty as well as results from a single-institution series of eight cases.

## Methods

This study was approved by an institutional review board (IRB STUDY00010248). The orthopedic surgical database at our institution was queried for patients who had undergone tendon transfer after shoulder arthroplasty procedure by the senior author (ADA). The inclusion criteria included those patients who had undergone a prior anatomic total shoulder arthroplasty, hemiarthroplasty, or resurfacing arthroplasty and subsequently presented with subscapularis insufficiency either clinically or radiographically or discovered at the time of revision surgery and were managed with surgical intervention using a pectoralis major tendon transfer by the senior author (ADA) between January 1, 2007, and June 30, 2020. Respective review of a medical record was performed for patient demographics, surgical procedural details, and clinical findings. All patients who underwent pectoralis major muscle transfer after anatomic total shoulder arthroplasty or hemiarthroplasty during this time frame were included in this review. Those who underwent pectoralis major tendon transfer for irreparable subscapularis tears in the setting of native joint surface and not in the setting of prior arthroplasty were excluded. There were 8 patients included in the review. Six patients had undergone prior anatomic total shoulder arthroplasty; 1 patient, a prior hemiarthroplasty; and 1 patient, a prior resurfacing arthroplasty that was revised to a total shoulder arthroplasty at time of pectoralis transfer.

## Indications

**Figure 1:**
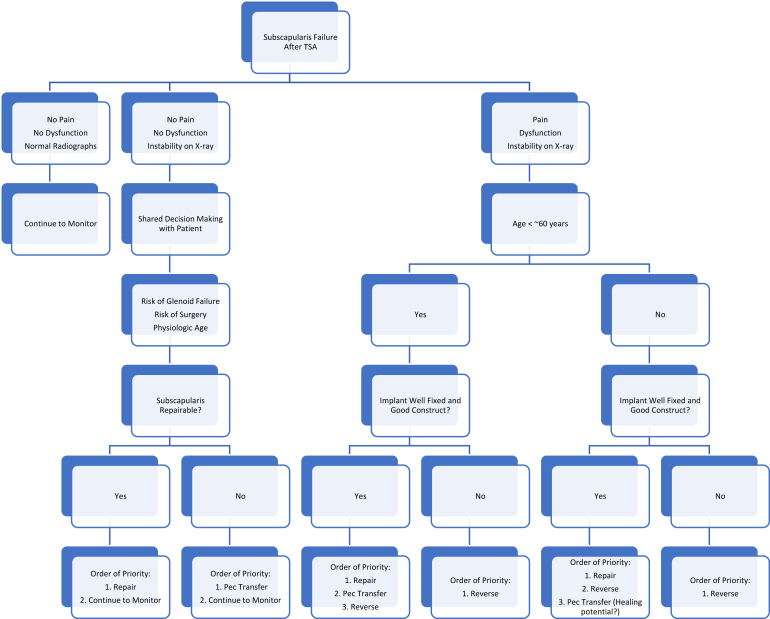
Decision-making flowchart for management of subscapularis insufficiency after anatomic total shoulder arthroplasty or hemiarthroplasty. *TSA*, total shoulder arthroplasty.

We propose the following decision guidelines for patients with suspected subscapularis insufficiency following anatomic shoulder arthroplasty (Fig. 1). Patients who were diagnosed with subscapularis insufficiency based on postoperative physical examination findings such as weakness with belly-press test, a positive belly-off test, or an abnormal lift-off test and were asymptomatic with no pain or dysfunction were managed nonoperatively. Those patients who were asymptomatic yet had radiographic changes such as anterior instability of the humeral head on the glenoid component on axillary images were considered for operative intervention. This was a shared decision-making model between the patient and surgeon. Surgical risk factors were considered; however, concern for eccentric load with the risk of early glenoid failure must also be weighed. Factors such as the degree of anterior instability demonstrated by the amount of translation on axillary images, as well as the physiological age and activity level of the patient, were taken into consideration. When discovered early, and in the setting of repairable tendon quality, primary repair of the subscapularis to the lesser tuberosity was recommended. In some cases, primary repair was not possible, dependent on the chronicity of the failure as well as the quality of the tissue. Additionally, as these patients were clinically asymptomatic, revision to reverse shoulder arthroplasty was not recommended due to the invasiveness of this procedure. In this case, a surgical discussion was again held with the patient regarding indications for reconstruction with pectoralis major tendon transfer vs. to continue nonoperative management with observation rehabilitation.

In the setting of patients with pain and dysfunction after anatomic total shoulder arthroplasty or hemiarthroplasty with anterior instability visualized on x-rays consistent with subscapularis insufficiency, operative management was recommended. In this scenario, the positioning and stability of the components is first determined. In the case of poorly placed components, such as overstuffing of the joint or loose components, primary repair of the subscapularis or tendon transfer may not be feasible. If the primary focus of the revision procedure is to decrease humeral head size alone but otherwise the position and stability of the components is adequate, then repair or reconstruction of the subscapularis with a tendon transfer may still be feasible. Otherwise, such as in the case to high stem positioning, revision of the components to a reverse shoulder arthroplasty would be preferred.

In patients with well-fixed components that are in good position and alignment, primary repair of subscapularis is recommended, when possible, with pectoralis major tendon transfer reserved for those cases in which the subscapularis is not repairable. Reverse shoulder arthroplasty may also be considered in this situation. This is the setting the physiological age and activity level of the patient must also be taken into consideration. In younger (<60 years) and more active individuals, we would recommend reconstructing the shoulder with a pectoralis major tendon transfer. In those patients with older physiological age or decreased functional demands and activity level, revision to reverse shoulder arthroplasty may be a better next step in treatment as to not to have to rely on the physiology of tendon healing.

One patient in our series had undergone an anatomic total shoulder arthroplasty by an outside surgeon in the setting of a prior brachial plexopathy. His main complaint on presentation was pain and instability. Though function was also decreased, the tendon transfer was not intended to restore function but rather to improve sensation of instability and discomfort.

## Surgical technique

**Figure 2:**
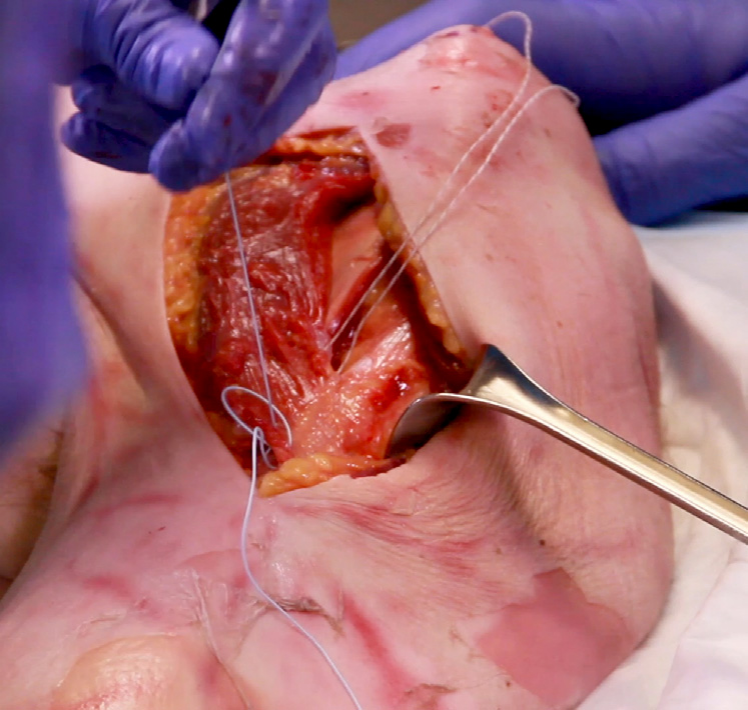
Clavicular and sternal heads of pectoralis tendon insertion dissected and tagged prior to release from humerus.

**Figure 3:**
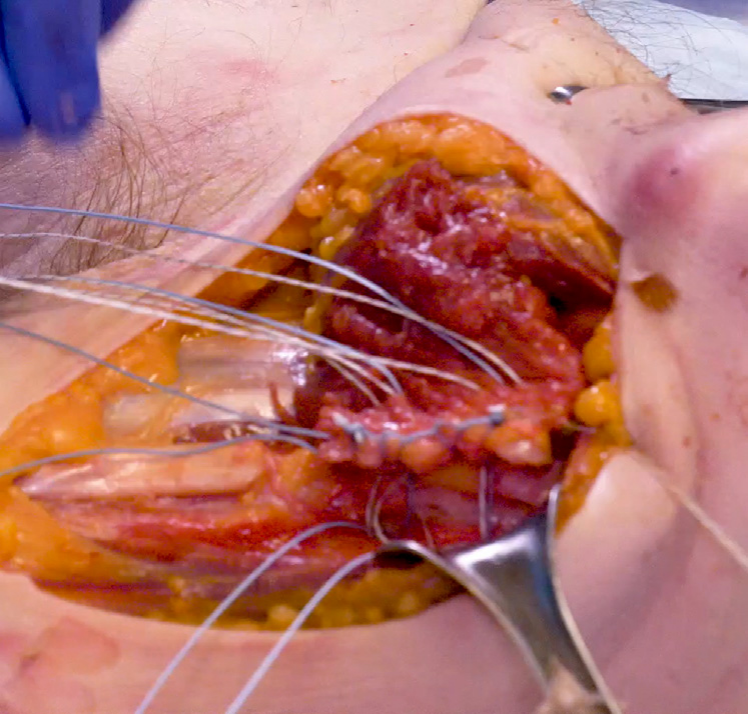
Pectoralis tendon with nonabsorbable suture in Kraków stitch on lateral tendon from superior to inferior. Sutures from anchors placed just lateral to the bicipital groove have been passed through the pectoralis tendon medial to the Kraków stitch in horizontal fashion.

**Figure 4:**
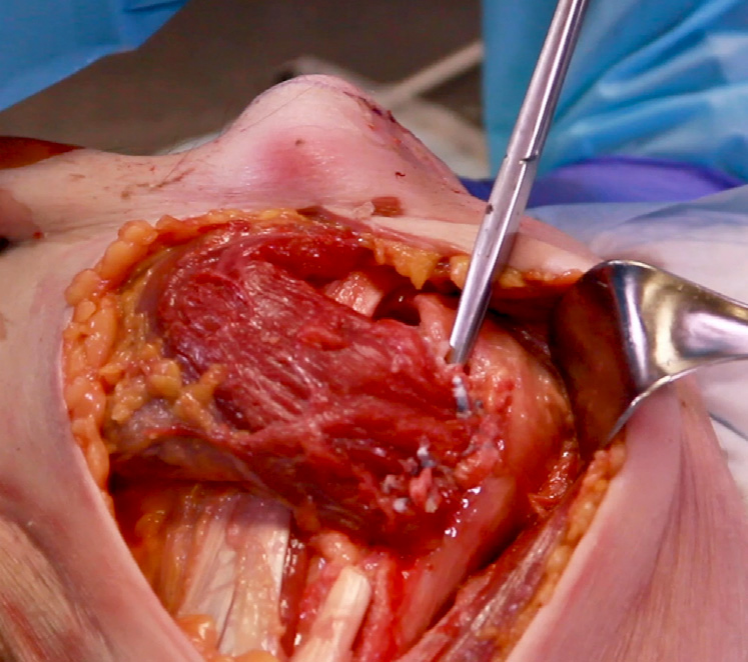
The sutures from the anchors have been tied completing the transfer. The Metzenbaum scissors are marking the bicipital groove. Notice the medial to lateral direction of the pectoralis when the transfer is placed lateral to the bicipital groove.

Deltopectoral exposure through the prior incision was utilized. Dissection was carried down to the deltopectoral interval. The deltoid was retracted laterally. The rotator cuff was then evaluated to ensure that the supraspinatus and infraspinatus remained intact. The subscapularis tendon rupture was then identified. This most often occurs at the superior aspect of the tendon. Mobilization with primary repair most often via suture anchor was then attempted. If the tear was not repairable and it was deemed that a pectoralis major tendon transfer was the next best option, then the focus was redirected to preparation of the pectoralis major tendon. The upper and lower border of the tendon was identified and bluntly dissected. The tendon was then sharply released directly from its humeral insertion. The entirety of the tendon was released from superior to inferior (Fig. 2). Both sternal and clavicular heads were transferred together being careful to not let the tendon twist on itself and keep the native orientation of the fibers. The transfer remained superficial to the conjoined tendon. The tendon was then mobilized, with care taken to avoid mobilizing further than 7 to 8 cm medial to protect the muscle innervation through the pectoral nerves. The tendon was then shifted superiorly while avoiding any twisting in the tendon. Two suture anchors were then placed in the anterior greater tuberosity (rather than the lesser tuberosity) directly lateral to the bicipital groove. This allowed for appropriate tensioning of the transfer by shifting both superior and lateral. This results in a more lateral to medial pull from the pectoralis. A nonabsorbable suture was used to place a locking Kraków stitch in the lateral aspect of the tendon from superior to inferior to prevent suture cutout. The sutures from the anchors were then placed through the tendon in horizontal mattress fashion and subsequently tied completing the transfer (Figs. 3 and 4). A second set of suture anchors may also be placed and utilized in simple fashion as a lateral row. The wound was then irrigated and closed in normal fashion. Postoperative protocol includes the use of a sling. External rotation was restricted to 20 degrees and progressively increased over the next few weeks. Anatomic total shoulder arthroplasty rehabilitation protocol was then utilized.

## Results

Eight patients met criteria for the review, including 6 anatomic total shoulder arthroplasties, 1 hemiarthroplasty (patient 6), and 1 resurfacing hemiarthroplasty (patient 5). Details of each case with age at time of pectoralis major tendon transfer are reported (Table I).

**Table I:** Outcomes of patients after pectoralis major tendon transfer.

Subject	Age	Sex	Type of arthroplasty	Additional surgery	Shoulder motion after transfer	Improved pain after transfer	Improved subjective stability	Radiographic alignment
1	65	F	TSA	Revision TSA	Regained	Yes	N/A	N/A
2	66	M	TSA	Reverse after fall	Regained	N/A	N/A	Temporary improvement
3	48	F	TSA		Temporarily improved	Yes	Yes	Temporary improvement
4	57	M	TSA		N/A (brachial plexopathy)	Yes	Temporary improvement	Temporary improvement
5	30	M	Resurfacing		Regained	Yes	Yes	Improved
6	39	F	HA		Regained	Yes	N/A	N/A
7	64	F	TSA	Reverse after fall	Temporarily improved	Yes	N/A	Temporary improvement
8	58	F	TSA		Regained	Yes	N/A	Temporary improvement

*TSA*, total shoulder arthroplasty.

Patient 1 was a 65-year-old woman with anatomic TSA before 1 year. She felt a subluxation event in shoulder and found to have a dislocated glenoid component, though no radiographic anterior instability was noted. She underwent removal of glenoid poly, bone grafting of glenoid defect, retained humeral components as hemiarthroplasty, and pectoralis transfer for subscapularis insufficiency found intraoperative. She was able to again regain her shoulder motion, and her pain initially improved. She experienced progression of the pain after approximately 1 year and ultimately underwent replacement of the glenoid component nearly 3 years after the pectoralis transfer procedure. The pectoralis transfer was intact at the time of that procedure and exposure was performed with a lesser tuberosity osteotomy. She again regained her shoulder motion and pain improved; however, she was found to have radiographic anterior instability after the lesser tuberosity osteotomy.

**Figure 5:**
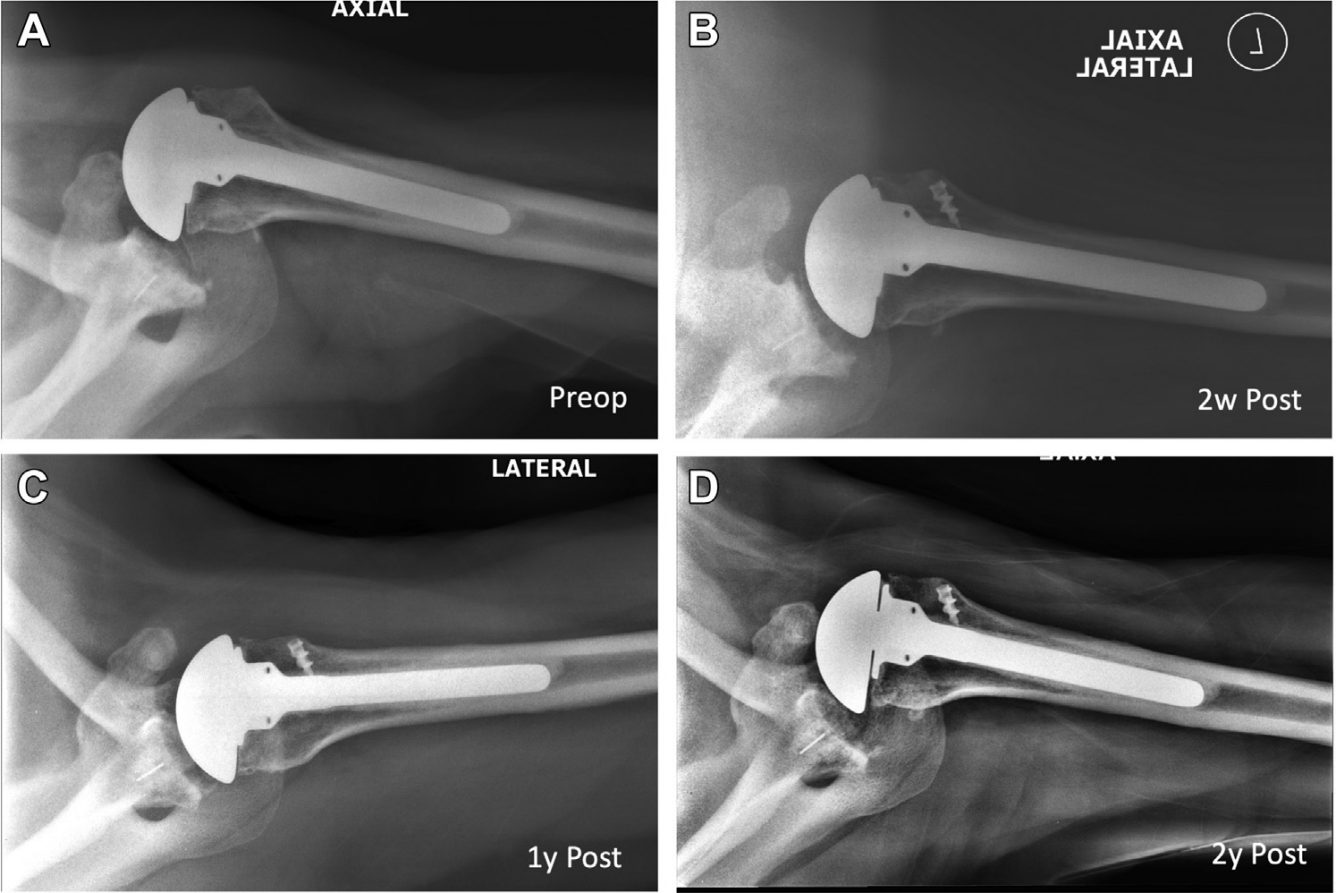
(**A**) Axillary shoulder radiographs from patient 2 demonstrating anterior translation of the humeral head on the glenoid preoperatively. (**B**) This alignment is improved on radiographs obtained 2 weeks postoperatively from pectoral major muscle transfer. (**C**) Continued alignment was noted at approximately 1 year postoperatively. (**D**) Anterior translation was discovered to recur on radiographs obtained approximately 2 years postoperatively. Patient continued to be pain free with preserved function despite radiographic instability.

Patient 2 was a 66-year-old man with anterior instability radiographically 6 weeks postoperatively. This progressed over the next year while he retained full motion without pain. At 16 months postoperatively, he underwent pectoralis tendon transfer with double-row technique. The radiographic instability improved initially but began to recur by 6 weeks postoperatively (Fig. 5). He continued with full motion and without pain until sustaining a fall 5 years later. He underwent revision to reverse total shoulder arthroplasty for massive rotator cuff tear at that time. There were sporadic ruptures of the transfer at the time of revision surgery.

Patient 3 was a 48-year-old woman with 5 prior surgeries including instability repair and resurfacing arthroplasty with revision subscapularis repair. She underwent revision to anatomic total shoulder arthroplasty with revision subscapularis repair. Postoperatively, she continued with pain, was unable to forward elevate at her shoulder, and had anterior translation radiographically. At 4 months postoperatively, she underwent a double-row pectoralis tendon transfer. Postoperatively, her pain improved, and she experienced near-full return of her shoulder active motion. Radiographic alignment improved temporarily; however, anterior translation recurred by 2 months. By 2 years postoperatively, she again had lost her active forward elevation, though pain was manageable and improved from that preoperatively.

Patient 4 was a 57-year-old man with brachial plexus injury in 1970s who underwent anatomic total shoulder arthroplasty by an outside surgeon presented 1 year postoperatively with pain, feeling of instability, and pseudoparalysis of the arm. He underwent a double-row pectoralis tendon transfer with the intent to improve his pain. Postoperatively, he continued with similar shoulder active motion as well as the sensation of instability; however, his shoulder pain had significantly improved. At nearly 10 years postoperatively, he continues with manageable shoulder pain that remains improved compared to that prior to the pectoralis transfer. He had anterior instability radiographically prior to the pectoralis transfer that improved temporarily postoperatively then recurred.

Patient 5 was a 30-year-old man with multiple prior instability procedures including open Bankart repair, taking narcotic pain medications for chronic pain. He underwent resurfacing arthroplasty and continued with pain, though slightly improved. He had near-full active motion and remained aligned radiographically. He continued with feeling of instability and progression of his pain with an ultrasound examination showing thinning of the subscapularis. He underwent a revision to total shoulder arthroplasty with a double-row pectoralis transfer. This resulted in significant improvement of his pain, and he continued with near-full active range of motion and radiographic alignment.

Patient 6 was a 39-year-old woman with a history of avascular necrosis treated with prior core decompression and subsequent hemiarthroplasty. She was utilizing narcotic medications for chronic pain. She experienced a fall 3 months postoperatively and was diagnosed with a subscapularis rupture. The subscapularis was found to be thin during the repair 6 months after the hemiarthroplasty. The tissue was repaired, and a pectoralis transfer was performed. Radiographically, the hemiarthroplasty remained aligned prior to the pectoralis transfer as well as after. Her shoulder motion after the tendon transfer was similar to that prior to the tendon transfer with forward elevation to 110°. Though she continued on chronic narcotics, she reported at 1 year postoperatively that her pain was significantly better than preoperative pain.

Patient 7 was a 64-year-old woman. Anatomic total shoulder arthroplasty was performed for post-traumatic arthritis from anterior-inferior glenoid fracture. The radiographs demonstrated anterior instability, and she continued with pain and subscapularis weakness, though had preserved forward elevation to 150°. She underwent revision repair of the minimal remaining subscapularis and double-row pectoralis transfer 2 years after the initial arthroplasty. Postoperatively, she was able to regain much of her active motion, had improvement in her pain, and demonstrated improved radiographic alignment. She had a recurrent injury to her shoulder at 7 months postoperatively resulting in increased pain, anterior radiographic translation, and loss of forward elevation. At 1 year postoperatively from the tendon transfer, she underwent revision to reverse total shoulder arthroplasty. At the time of the revision arthroplasty, the upper fibers of the pectoralis tendon transfer were found to be ruptured. The remining intact tissue was pealed from the humerus for exposure. This tissue was repaired again after placement of the reverse components.

Patient 8 was a 58-year-old woman with stemless total shoulder arthroplasty. She experienced progressive pain and demonstrated anterior translation radiographically. She underwent partial subscapularis repair with double-row pectoralis transfer a year and a half after the initial arthroplasty. She had preserved forward elevation prior to tendon transfer that she was able to regain postoperatively. The radiographs showed temporary improvement in alignment with slight recurrence of the anterior translation at 4 months postoperatively. Her pain had improved from that prior to the tendon transfer.

## Discussion

Pectoralis muscle transfer can improve pain and subjective instability, while preserving function in physiological young patients with subscapularis failure after anatomic shoulder arthroplasty in the setting of well-positioned and well-fixed components.

Management of patients with subscapularis insufficiency after anatomic total shoulder arthroplasty or hemiarthroplasty can be challenging. In cases where the patient is asymptomatic, though physical examination suggests subscapularis failure as demonstrated with belly-press testing, nonoperative management with rehabilitation can be considered. Long-term sequela of this is unknown as there is a concern for glenoid component loosening with continued instability.^16^^,^^17^ When radiographic instability is identified, surgical intervention is often recommended to decrease the risk of component failure. When primary repair of the subscapularis to the lesser tuberosity is possible, this is clearly the recommended treatment of choice. In patients where primary repair is not achievable, either due to chronicity of the tear, retraction of the tendon or quality of the tissues, pectoralis major tendon transfer vs. revision to reverse shoulder arthroplasty may be considered.

Entezari et al^4^ reviewed 25 patients who demonstrated symptomatic subscapularis failure after anatomic total shoulder arthroplasty and underwent either revision subscapularis repair or revision to reverse shoulder arthroplasty. Decision-making on management strategy was related to patient age, activity level, medical comorbidities, timing, and mechanism of subscapularis failure. Patients who underwent subscapularis repair were significantly younger and had a better comorbidity profile with a more acute presentation. They also demonstrate a higher reoperation rate than revision to reverse shoulder arthroplasty. Two of the 9 cases were ultimately revised to pectoralis major tendon transfers. Patient-reported outcome and functional scores were not significantly different between the groups.

Pectoralis major tendon transfer is a well-described surgical management for reconstruction of irreparable rotator cuff repairs of the anterior and anterior superior rotator cuff.^11^ Techniques that transfer the pectoralis major in its entirety as well as splitting of the tendon have been described.^7^ Split transfers of both the sternal head and clavicular head in isolation have both been described.^8^^,^^22^ The tendon can be rerouted beneath the conjoined tendon or superficially to lie over the conjoined tendon.^18^ Tendon transfer deep to the conjoined tendon is thought to provide some additional relief of subcoracoid impingement through a soft-tissue interposition effect. The surgeon must be cautious however of the musculocutaneous nerve lying deep to the conjoined tendon. In the case of subconjoined transfer, the transferred pectoralis tendon should be placed superficial to the musculocutaneous nerve though deep to the conjoined tendon, as placing the transfer deep to the musculocutaneous nerve may lead to increased tension on the nerve.^6^^,^^14^^,^^22^ In this subconjoined transfer, split-tendon transfers are often recommended to decrease the risk to the musculocutaneous nerve.^14^^,^^18^

When routing the pectoralis tendon superficial to the conjoint tendon, there are some key surgical techniques and considerations. Some key concepts from the author's preferred technique for pectoralis major tendon transfer superficial to the conjoint tendon are the following: (1) The pectoralis major tendon in its entirety was transferred including both the sternal and clavicular heads and being careful to not allow the tendon to twist on itself. (2) The tendon is transferred superficial to the conjoined tendon but placed too proximal onto the humeral head in order to create a more direct compressive line of action from lateral to medial. (3) The pectoralis major tendon is transferred lateral to the bicipital groove to accommodate the redundancy of the tendon. When transferred medial to the bicipital groove, there is often laxity to the tendon. The reasoning behind these technical points is to increase the bulk of the tendon that is transferred, avoid additional dissection and risk to the musculocutaneous nerve, and that transfer of the pectoralis major tendon lateral to the bicipital grove has been demonstrated to better restore muscle length-tension relationship.^14^

Though favorable results have been demonstrated after pectoralis major tendon transfer for irreparable subscapularis tendon tears, the results of pectoralis major tendon transfer in the setting of prior arthroplasty are less well described.^5^ In a study by Elhassan et al,^3^ 8patients who had previously undergone shoulder arthroplasty, including 5 anatomic total shoulder arthroplasties and 3 hemiarthroplasties, were treated with split subconjoined sternal head pectoralis major muscle transfer. The results were overall poor with only 1 patient reporting significant improvement in pain and subjective score, and the belly-press test remaining positive in all patients. The mean pain score improved slightly, though not statistically significant, which was similar to the improvement in the Constant score, also not statistically significant. Six patients reported no improvement in pain or function, and rupture of the transfer was demonstrated on computed tomography arthrogram in all 6. Shin et al^25^ also reported less-favorable functional outcomes after pectoralis major transfer and patients with prior shoulder arthroplasty in their systematic review.

Our series of 8 patients demonstrated more favorable results with pectoralis major tendon transfer after shoulder arthroplasty. These benefits include improvement of pain, preservation of function, and improvement of subjective feeling of instability. Improvement of pain was noted in all patients (7 patients) who were experiencing preoperative discomfort. Though pain was not completely resolved in most patients, significant improvement was reported. Additionally, the subjective improvement of the preoperative feeling of instability of the shoulder was improved in all cases where instability sensation was present (3 patients).

Those patient with preserved shoulder function with forward elevation prior to the pectoralis transfer were able to regain similar forward elevation after the transfer. One patient with a brachial plexus palsy was not expected to make functional improvements (patient 4). Another patient with poor preoperative, active forward elevation did not improve postoperatively; however, this patient did temporarily regain active motion, but this was only maintained for approximately 2 years until loss of active motion was again present (patient 3). Of the 6 patients with preserved motion preoperatively, 5 were able to return to similar function except for 1 patient who fell at 7 months postoperatively and had only partial return of motion up until the time of the fall (patient 7.)

Radiographic improvement of humeral head alignment on the glenoid was temporary in most cases. Six of the patients had anterior translation of the humerus on the glenoid preoperatively. All 6 patients were shown to have correction of the translation of initial postoperative radiographs. However, in 5 of those patients, the radiographic translation recurred at variable timeframes postoperatively. It is unclear why the radiographic changes were lot as long-lasting, though it is important to note that the radiographic changes did not correlate with a recurrence of pain, worsening function, or recurrence of subjective instability. One patient was not painful preoperatively but found to have radiographic instability only.

We suspect the better outcomes in this series may be related to the increased bulk of the transfer to provide stability to the joint and aligning the transfer to encourage a direct lateral to medial line of action. Furthermore, transfer of the tendon not to the lesser tuberosity, but rather to the anterior greater tuberosity directly lateral to the bicipital groove better restores the muscle length-tension relationship and perhaps leads to improved outcomes. It is also possible that the transfer has a tenodesis effect. The tendon was placed superficial to the conjoined tendon due to the bulk of the transfer and in order to avoid injury to the musculocutaneous nerve.

We recognize this study has limitation. Most notable is the nature of a retrospective review leading to error and incompleteness of the data available to be collected from the medical record. Additionally, we recognize that 8 patients provide only a small sample size, though this number is similar to prior reports of this uncommon procedure. These data were also collected over many years and do not take into account evolution of changes in surgical technique and postoperative management. Lastly, this patient group, though all had undergone shoulder arthroplasty and sustained failure of the subscapularis, presented as a very heterogenous population. Some patients had undergone multiple prior surgeries, while others, only a primary anatomic total shoulder arthroplasty, in addition to the variety of other medical comorbidities that cannot be accounted for in this study.

## Conclusion

Failure of subscapularis healing after total shoulder arthroplasty can result in pain, dysfunction, and instability. Operative management is indicated to manage pain, preserve or restore function, and provide stability. Pectoralis muscle transfer may be considered in physiological young patients with an irreparable subscapularis who have well-positioned and well-fixed components. Pectoralis major muscle transfer can improve pain and subjective feeling of instability while preserving motion. Though radiographic instability often improved early, these benefits were often temporary. However, patients continued clinically do well. Pectoralis transfer avoids a more-invasive approach in the case of revision reverse total shoulder arthroplasty, though reverse shoulder arthroplasty may be a better and more reliable alternative in physiological older patients if there is concern for healing potential of the tendon transfer. Those patient with pseudoparalysis may benefit more reliably from revision to reverse total shoulder arthroplasty, though patients with preserved active motion are often able to retain it. Pectoralis major tendon transfer has potential to improve pain and preserve function while still allowing for the opportunity to revise to reverse total shoulder arthroplasty in the future. Pectoralis major tendon transfer is considered a good salvage and less-invasive surgical option, compared to revision reverse shoulder arthroplasty, for younger patients.

## Disclaimers:

Funding: No funding was disclosed by the authors.

Conflicts of interest: Dr. Updegrove has served as a consultant to Aevumed. Dr. Armstrong has served as a consultant to Zimmer Biomet, Globus, and Aevumed. The other authors, their immediate families, and any research foundation with which they are affiliated have not received any financial payments or other benefits from any commercial entity related to the subject of this article.
